# Comparative epidemiological study of breast cancer in humans and canine mammary tumors: insights from Portugal

**DOI:** 10.3389/fvets.2023.1271097

**Published:** 2023-11-30

**Authors:** Paulo Tiago Carvalho, João Niza-Ribeiro, Irina Amorim, Felisbina Queiroga, Milton Severo, Ana Isabel Ribeiro, Katia Pinello

**Affiliations:** ^1^Vet-OncoNet, Population Studies Department, School of Medicine and Biomedical Sciences, ICBAS, University of Porto, Porto, Portugal; ^2^Epidemiology Unit (EPIUnit), Institute of Public Health of the University of Porto (ISPUP), Porto, Portugal; ^3^Laboratory for Integrative and Translational Research in Population Health (ITR), Porto, Portugal; ^4^Department of Pathology and Molecular Immunology, School of Medicine and Biomedical Sciences ICBAS, University of Porto, Porto, Portugal; ^5^Department of Veterinary Sciences, University Trás-os-Montes and Alto Douro (UTAD), Vila Real, Portugal; ^6^Centre for Animal and Veterinary Science (CECAV), University of Trás-os-Montes and Alto Douro, Vila Real, Portugal; ^7^Center for Animal Science Studies, Institute of Sciences, Technologies and Agroenvironment (CECA-ICETA), University of Porto, Porto, Portugal; ^8^Department of Public Health and Forensic Sciences and Medical Education, Faculty of Medicine, University of Porto, Porto, Portugal

**Keywords:** breast cancer, canine, cluster analysis, comparative oncology, environmental, LISA cluster analysis, geographic, mammary gland tumor

## Abstract

Dogs spontaneously develop mammary gland tumors (MGT) and exhibit striking similarities in clinical and epidemiological characteristics to human breast cancer (HBC). Descriptive and comparative analysis of HBC and canine MGT with a focus on evaluating similarities and geographical distribution were the aims of this study. HBC cases were obtained from North Regional Oncological Registry (RORENO) (2010–2015) and canine MGT cases from Vet-OncoNet (2019–2022). Analyses were performed based on published and well accepted classification systems (ICD-O-3.2 for humans and Vet-ICD-O-canine-1). Age-standardized incidence risks (ASIR) of Porto district municipalities were calculated using 2021 Portuguese census (INE) and data from the Portuguese animal registration system (SIAC). Among 7,674 HBC cases and 1,140 MGT cases, a similar age and sex distribution pattern was observed. Approximately 69.2% of HBC cases were between 40 and 69 years old, while 66.9% of MGT cases were diagnosed between 7 and 12 years old (mean age of 9.6 years, SD = 2.6). In women, Invasive breast carcinoma (8500/3) was the most common histological type (*n* = 5,679, 74%) while in dogs it was the Complex Carcinoma (8983.1/3) (*n* = 205, 39%). Cocker and Yorkshire Terriers exhibited the highest relative risks (3.2 and 1.6, *p* < 0.05, respectively) when compared to cross breed dogs. The municipalities' ASIR of the two species exhibited a high correlation (R = 0.85, *p* < 0.01) and the spatial cluster analysis revealed similar geographic hotspots. Also, higher ASIR values both in women and dogs were more frequently found in urbanized areas compared to rural areas. This research sheds light on the shared features and geographical correlation between HBC and canine MGT, highlighting the potential of cross-species environmental oncology studies.

## 1 Introduction

Breast cancer is the most commonly diagnosed cancer in women worldwide (1). In 2018, there were approximately 2.1 million newly diagnosed cases of women's breast cancer (WBC), accounting for one in four cancer cases among women and leading to approximately 630,000 deaths (2). In Portugal, breast cancer has a significant impact, with an incidence rate of 139.14 per 100,000 women (3).

Breast cancer encompasses various subtypes with distinct molecular and cellular origins, as well as differing clinical behaviors (4). The two most prevalent subtypes are invasive carcinoma of the breast, not otherwise specified (NOS), which accounts for 70 to 75% of cases, and lobular carcinoma, comprising 12 to 15% of cases (4). Additionally, there are 18 rare subtypes (ranging from 0.5 to 5%), each displaying specific morphological features (5). In Portugal, breast cancer ranks highest among women in the latest Portuguese National Cancer Registry (6). The Portuguese National Cancer Registry (RON) centrally registers all cancer cases via an electronic platform. RORENO, the Northern Region-specific registry collects data on new tumor cases among Northern Region residents.

Mammary gland tumors (MGT) are highly prevalent in dogs ranking as the second most common tumor (7–9). Canine mammary carcinomas have an estimated annual incidence of 192 cases per 100,000 female dogs (3, 10, 11), with 41% to 53% of these tumors being malignant (12). However, the histologic evidence of malignancy does not always indicate a malignant clinical course, and there can be significant histologic variation within the same tumor (5). The majority of malignant mammary tumors in dogs are classified as epithelial tumors or carcinomas, while pure sarcomas are less common (13, 14).

To facilitate global registration and comparison of animal cancer cases, the Global Initiative for Veterinary Cancer Surveillance (GIVCS) was established, leading to the development of a comparative coding system for canine tumors called Vet-ICD-O-canine-1 (15). This system aligns with the human classification ICD-O-3.2, enabling comparability between veterinary and human cancer registries. Unique to dogs, the Vet-ICD-O-canine-1 includes specific veterinary codes for each canine mammary gland [cranial thoracic (C50.10), caudal thoracic (C50/20), cranial abdominal (C50.30), caudal abdominal (C50.40), and inguinal (C50.50)] and also for specific morphologies as carcinoma arising in a complex adenoma/benign mixed tumor (8941.1/3) and complex carcinoma (8983.1/3) (15).

In Portugal, as per the latest Animal Cancer Registry (ACR) from 2021, mammary gland tumors are the most common among female dogs (16). Published by Vet-OncoNet, the ACR compiles data from a vast network of veterinary laboratories across the country, encompassing 75% of all animal cancer cases in Portugal (17).

Recent advances in comparative oncology have emphasized the value of dogs as a valuable model for the spontaneous development of cancer in humans, enabling the scientific community to develop preventive approaches (18–20). Naturally occurring canine MGT share numerous similarities with their human counterparts, including incidence, age of onset, risk factors, biological behavior, metastatic patterns, histological, molecular, and genetic features, as well as therapy response (21–24).

Age is the most significant known risk factor for breast cancer in women (WBC), with a peak incidence at menopause and a subsequent gradual decrease or stability (1, 2). Less than 5% of cancer cases in humans occur before the age of 35, and <10% occur before the age of 45 (25). A similar pattern is observed in dogs, where <5 to 10% of cancer cases are diagnosed between birth and 3–5 years (26). The increased longevity of companion animals has contributed to the rise in cancer diagnoses (27).

Human breast cancer incidence and mortality rates vary across geographic regions and ethnic populations (28). African American women have a lower incidence of breast cancer compared to White women but experience higher overall mortality rates (29). Incidence and mortality rates also vary among Asian and Hispanic/Latino populations (30, 31). Similarly, the incidence of mammary tumors in dogs varies by breed. Although the specific high-risk breeds differ between studies and geographic locations (32). Poodles, Chihuahuas, Dachshunds, Yorkshire Terriers, Maltese, and Cocker Spaniels are frequently listed as high-risk small breed dogs, while larger breeds such as English Springer Spaniels, English Setters, Brittany Spaniels, German Shepherds, Pointers, Doberman Pinschers, and Boxers are also at higher risk (32, 33).

In women, the age of menopause and certain reproductive factors are associated with an increased risk of breast cancer development (1, 34). Similarly, the endocrine environment, defined by exposure to estrogen and progesterone, plays a role in the development of canine mammary carcinomas (35–37). Ovariohysterectomy or ovariectomy performed before the first estrus greatly reduces the risk of developing mammary tumors in female dogs, with the risk decreasing to 0.5, 8, and 26% if the surgery is performed before the first, second, or after the second estrus, respectively (38). Moreover, the incidence by sex is also similar in both species. Breast cancer is more commonly observed in women compared to men, with <1% of all human cancer cases occurring in men (39). Similarly, MGT in dogs follows a similar trend, being rare in male dogs, with a risk approximately 62 times lower than that in female dogs (7, 40).

Genetic predisposition is well-established in human breast cancer, with certain genes and mutations associated with increased risk (41, 42). Dogs also show a genetic predisposition to develop mammary tumors, with specific breed-related associations and the presence of risk alleles linked to heritable cancer risk (43). Genetic similarities between human and canine cancers have been observed, including the presence of BRCA1/BRCA2 germline mutations (44, 45). Epigenetic mechanisms, which can influence the development of different phenotypic identities, are also being studied in both human and canine breast cancers (46–48).

Environmental factors, including air pollution and persistent organic pollutants (POPs), have been linked to breast cancer incidence (49, 50). Studies have shown a positive correlation between air pollution and postmenopausal breast cancer rates in European women (49). Dogs sharing environments with humans, can be affected by these environmental pollutants and serve as biosentinels for human exposure to contaminants (50, 51).

The remarkable similarities in epidemiology and clinicopathological characteristics between spontaneous tumors in companion animals and their human counterparts and shared exposure to similar risk factors have positioned companion animals as valuable models in human cancer research. Companion animals, such as cats and dogs, progress through cancer at a faster rate than humans due to their shorter lifespan. These unique aspects enable the study of cancer in companion animals to provide crucial insights for comparative oncology (52). Therefore, the objective of this study is to conduct a descriptive and comparative analysis of human breast cancer and canine mammary gland tumors in the Porto district in Northern Portugal, with a focus on evaluating epidemiological similarities, geographical distribution, and potential associations.

## 2 Materials and methods

### 2.1 Study design

This retrospective cohort study compares the distributions and incidence risks of breast cancer in humans and mammary gland tumors in dogs within the Porto district, Portugal.

### 2.2 Data collection

Human Breast Cancer (HBC) cases were obtained upon request from the North regional cancer registry (RORENO) between 2010 and 2015, specifically for the Porto district. The collected data included sex, age group, municipality, date of diagnosis, tumor topography (C50.-), morphology (histological type), and tumor behavior (/3), categorized according to the International Classification of Diseases for Oncology, 3rd edition (ICD-O-3.2). Data on the human population were obtained from the Portuguese National Institute of Statistics, based on the 2021 census.

Canine mammary gland tumor (MGT) cases were collected from the Vet-OncoNet platform between 2019 and 2022. The veterinary laboratory partners (VLP), including DNATech, Cedivet, Veterinary Pathology Laboratory – VetPat, Segalab, Laboratory of Veterinary Pathology at the University of Lisbon, and the Laboratory of Veterinary Pathology at the University of Porto, provided the data. The collected information included age, sex, breed, postal code of the requesting veterinary centers, date of diagnosis, tumor topography (C50.-), morphology (histological type), and tumor behavior (/3), categorized according to the Veterinary International Classification of Diseases for Oncology-canine (Vet-ICD-O-canine), 1st edition (15). Data on the canine population were obtained from the Portuguese Companion Animal Information System (SIAC)^1^, the official site for the compulsive registry of domestic animals, which provides the national census for domestic animals.

### 2.3 Data analysis

After performing internal validity checks and data cleaning using Microsoft Office Excel 2013, the analysis was conducted using Excel and R, version 4.1.2. Categorical variables were presented as counts and percentages and analyzed using Z-tests. A Z-test against 50% (50% Z-test) was performed to determine if the proportion of cases differed significantly from the expected balanced data between sexes. Continuous variables were expressed as mean and standard deviation or median and interquartile range (IQR). Differences in mean age were assessed using Student's *t-*test for two categories and ANOVA followed by the Tukey test for three or more categories.

Relative Risk (RR) was calculated for each breed per benign, malignant, and all MGT cases, comparing the cases to the breed population in SIAC. Proportional differences were assessed using chi-square tests, and 95% confidence intervals were computed. No-breed dogs were used as the reference group. The comparative analysis between humans breast cancer and canine mammary gland tumors included only malignant MGT cases (mMGT).

A Z-score was calculated to allow a comparative analysis of the ages between HBC and canine MGT, using the median value of each age range for HBC. Wilcoxon rank-sum tests were performed to determine differences in the medians of the Z-scores, and an F-test was conducted for variances. Two-sample Kolmogorov-Smirnov tests were also performed to compare the distributions of the Z-scores.

To determine the geographic distribution of WBC and female dogs with mMGT in the Porto district, data from the women population available on the INE website (Supplementary Table S4) and the population of female dogs from SIAC (Supplementary Table S5) were considered.

Women and female dogs' age-standardized incidence risks (ASIR) were calculated for both species by taking into account the number of cases and the corresponding population per municipality. This calculation was performed over the specific data period and multiplied by 10,000 for ease of interpretation. The direct standardized method was employed, using the human and canine populations of Porto District as the reference.

Spatial analysis was conducted in GeoDa version 1.20 and mapping was conducted in QGIS 3.22.13. GeoDa was used to measure the global spatial autocorrelation using Moran's Index (Moran's I) and detect local clusters using Local Index of Spatial Autocorrelation (LISA). The global Moran's I statistic was used to measure the overall degree of clustering. A global Moran's I > 0 indicates a clustered pattern (i.e., similar values are found near each other), I = 0 indicates a random pattern, and I < 0 indicates a dispersed pattern. Then, the LISA was calculated for each municipality in the study area to identify spatial clusters of similar cancer rates. It allows to identify whether a municipality was part of a cluster of similar values (high-high or low-low) or if it was surrounded by dissimilar values (high-low or low-high). Additionally, we also calculated the Bivariate Moran's to examine the spatial relationship between the rates of WBC and mMGT, simultaneously. It determines whether there is a spatial association between the values of the two variables, helping to identify patterns of similarity in their geographical distribution.

Pearson correlation coefficient (R) was calculated between the ASIR of women and female dogs per municipality, along with the respective *p-*value. A cluster analysis of the correlation points by municipalities was performed using the R (library NbClust), with Euclidean distance and the single method.

All statistical analyses were conducted using R, and statistically significant results were reported at *p* < 0.05 for two-sided tests.

This study was approved by the Animal Welfare Ethics Committee (ORBEA) of the School of Medicine and Biomedical Sciences–ICBAS, University of Porto (P310/2019/ORBEA) and is part of the Vet-OncoNet project. Data from human cases were obtained through a request to the Ethics Committee of the Portuguese Institute of Oncology (IPO) in Porto with reference 160/2021.

## 3 Results

### 3.1 Breast cancer

A total of 7,674 breast cancer (BC) cases from the Porto district were included in this analysis, covering the period from January 2010 to December 2015. In terms of sex distribution, Table 1 reveals a predominance of women, accounting for 98.8% of the cases (*n* = 7,584), while men represented only 1.2% (*n* = 90) of the cases. Looking at the age distribution, Table 1 shows that 60.6% of the cases occurred in women between the ages of 45 and 69. The most frequent registered topography for breast cancer was Breast, NOS (C50.9), accounting for 27.2% of the cases, followed by overlapping lesions (C50.8) (Table 1). Regarding the distribution of morphologies (Table 1), the Invasive breast carcinoma of no special type (former Infiltrating Duct Carcinoma, NOS (8500/3) was the most commonly observed type in breast cancer cases, accounting for 74% of the cases (*n* = 5,679), followed by Lobular Carcinoma, NOS (8520/3) with 9.2% (*n* = 707) of the cases.

**Table 1:** Descriptive analysis of human breast cancer registered between 2010 and 2015 in the Porto District, Portugal.

	***n***	**%**
**Sex**		
Female	7,584	98.8
Male	90	1.2
**Age (years)**		
20–44	1,217	15.9
45–69	4,654	60.6
>70	1,765	23.0
**Topography**_(ICD − O−3.2)_		
Nipple _(C50.0)_	44	0.6
Central portion _(C50.1)_	288	3.8
Upper-inner quadrant _(C50.2)_	632	8.2
Lower-inner quadrante _(C50.3)_	278	3.6
Upper-outer quadrant _(C50.4)_	1,904	24.8
Lower-outer quadrante _(C50.5)_	345	4.5
Axillary tail _(C50.6)_	18	0.2
Overlapping lesion _(C50.8)_	2,078	27.1
Breast, NOS _(C50.9)_	2,087	27.2
**Morphologies**_(ICD − O−3.2)_		
Invasive breast carcinoma of no special type^*^_(8500/3)_	5,679	74.0
Lobular carcinoma, NOS _(8520/3)_	707	9.2
Infiltrating duct mixed with other types of carcinomas _(8523/3)_	345	4.5
Infiltrating duct and lobular carcinoma _(8522/3)_	204	2.7
Carcinoma, NOS _(8010/3)_	165	2.2
Mucinous adenocarcinoma _(8480/3)_	105	1.4
Neoplasm, malignant _(8000/3)_	99	1.3
Infiltrating ductular carcinoma _(8521/3)_	57	0.7
Invasive micropapillary carcinoma of breast _(8507/3)_	42	0.5
Intraductal papillary adenocarcinoma with invasion _(8503/3)_	36	0.5
Metaplastic carcinoma, NOS _(8575/3)_	31	0.4
Tubular adenocarcinoma _(8211/3)_	28	0.4
Paget disease, mammary _(8540/3)_	23	0.3
Encapsulated papillary carcinoma with invasion _(8504/3)_	20	0.3
Others	133	0.7

Data from the North Regional Oncological Registry (RORENO). ^*^former Infiltrating duct carcinoma, NOS.

### 3.2 Mammary gland tumors

A total of 1,140 mammary gland tumors (MGT) were registered in the Vet-OncoNet from the Porto District. Among these, 1,115 cases were observed in female dogs, accounting for 97.8% of the total, while 25 cases were found in male dogs, representing 2.2% (Table 2). The mean age of dogs diagnosed with MGT was 9.6 years (SD = 2.6). Female dogs had a significantly higher mean age (9.7 years, SD = 2.6) compared to male dogs (8.0 years, SD = 4.0) (*t-*test = 3.1, *p* = 0.001).

**Table 2:** Descriptive analysis of mammary gland tumors in dogs registered at Vet-OncoNet database, between 2019 and 2022.

	**Benign tumors**		**Malignant tumors**	
	***n***	**%**	***n***	**%**
Total _(n = 1, 140)_	605	53.1	535	46.9
**Sex**				
Female _(n = 1, 115, 97.8%)_	588	52.7	527	47.3
Male _(n = 25, 2.2%)_	17	68.0	8	32.0
**Age**				
0–6 _(n = 117, 10.3%)_	68	58.1	49	41.9
7–11 _(n = 640, 56.1%)_	350	54.7	290	45.3
>12 _(n = 259, 22.7%)_	120	46.3	139	53.7
Missings _(n = 124, 10.9%)_	67	54.0	58	46.0
**Morphology**_(Vet − ICD − O)_	(*n* > 10)			
Complex adenoma _(8983/0)_	220	19.3	-	-
Benign mixed tumor, NOS _(8940/0)_	218	19.1	-	-
Complex carcinoma _(8983.1/3)_	-	-	205	18.0
Tubular carcinoma _(8211/3)_	-	-	119	10.4
Tubular adenoma _(8211/0)_	113	9.9	-	-
Solid carcinoma _(8230/3)_	-	-	76	6.7
Tubulopapillary carcinoma _(8263/3)_	-	-	46	4.0
Intraductal papillary adenoma _(8503/0)_	16	1.4	-	-
Carcinosarcoma, NOS _(8980/3)_	-	-	15	1.3
Invasive micropapillary carcinoma _(8507/3)_	-	-	10	0.9
Others	37	3.2	65	5.7

More than half of the cases were classified as benign tumors (Table 2), which showed a significantly lower mean age compared to malignant tumors (9.4 years, SD = 2.6 vs. 9.9 years, SD = 2.6) (*t*-test = 3.2, *p* = 0.001). In terms of topography, the majority of cases (95.5%, *n* = 1,103) were classified as Mammary gland, NOS (C50.9).

The most frequent morphologies observed were complex adenoma, accounting for 19.3% (*n* = 220) of the cases, followed by benign mixed tumor (*n* = 218, 19.1%) and complex carcinoma with 18.0% (*n* = 205) (Table 2).

**Figure 1:**
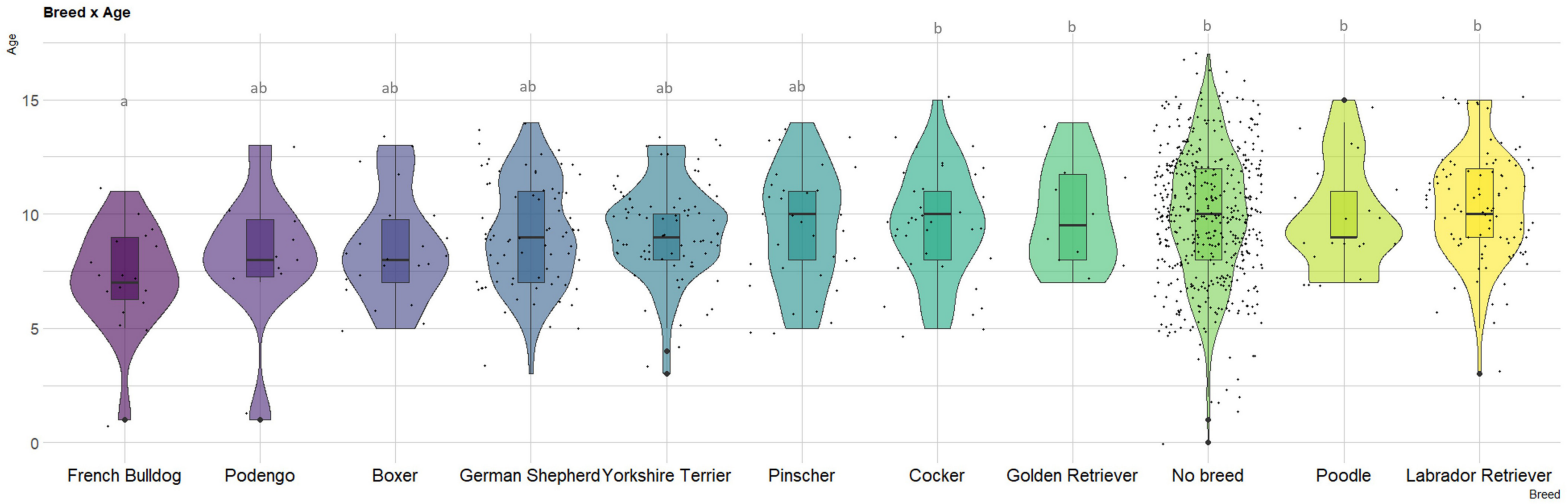
Violin plot depicting age at canine mammary gland tumor (MGT) diagnosis among major breeds (*n* > 10). The letters represent differences in the mean ages as determined by the Tukey test.

In terms of breed distribution among dogs with mammary gland tumors (Supplementary Table S1), the most affected group consisted of no-breed dogs (*n* = 572, 50.2%), followed by Labrador Retrievers (*n* = 103, 9.0%), Yorkshire Terriers (*n* = 88, 7.7%), and German Shepherds (*n* = 86, 7.6%). Analyzing the age distribution (Supplementary Table S1, Figure 1), Labrador Retrievers exhibited the highest mean age (10.4 years, SD = 2.4), while French Bulldogs had the lowest mean age (7.2 years, SD = 2.3), being these differences statistically significant.

**Figure 2:**
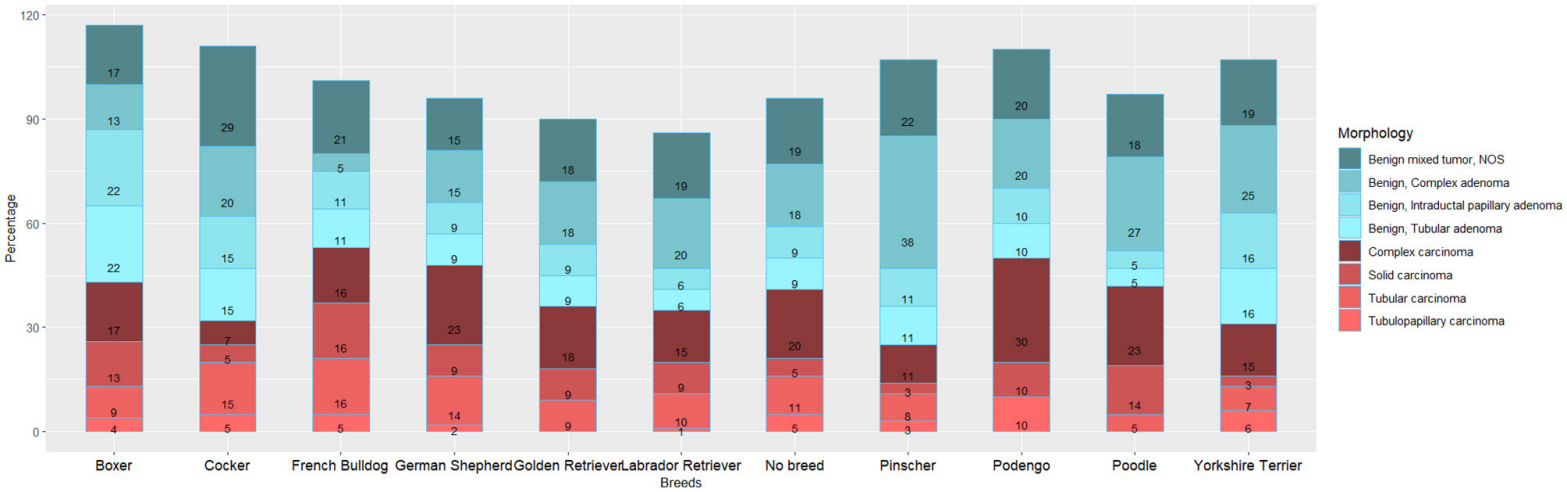
Distribution of canine mammary gland tumor (MGT) morphologies by major breeds (*n* > 10).

The analysis of the distribution of the most frequent morphologies by the main breeds (with *n* > 10) (Figure 2) reveals slightly different patterns across breeds. French Bulldogs shows a higher proportion of malignant tumors, while Pinschers and Yorkshire Terriers exhibit a lower proportion. Among Cockers, there is a higher proportion of Benign Mixed tumors (29%), whereas Podengos have a higher proportion of complex carcinomas (29%), and Pinschers show a higher proportion of complex adenomas (38%).

Regarding the proportion of malignancy (PM), the results indicate that non-breed dogs have a higher PM (48.3%) compared to purebred dogs (45.7%). Among the breeds, French Bulldogs and German Shepherds exhibit the highest MP, whereas Pinschers and Yorkshire Terriers have lower proportions (Figure 2).

**Figure 3:**
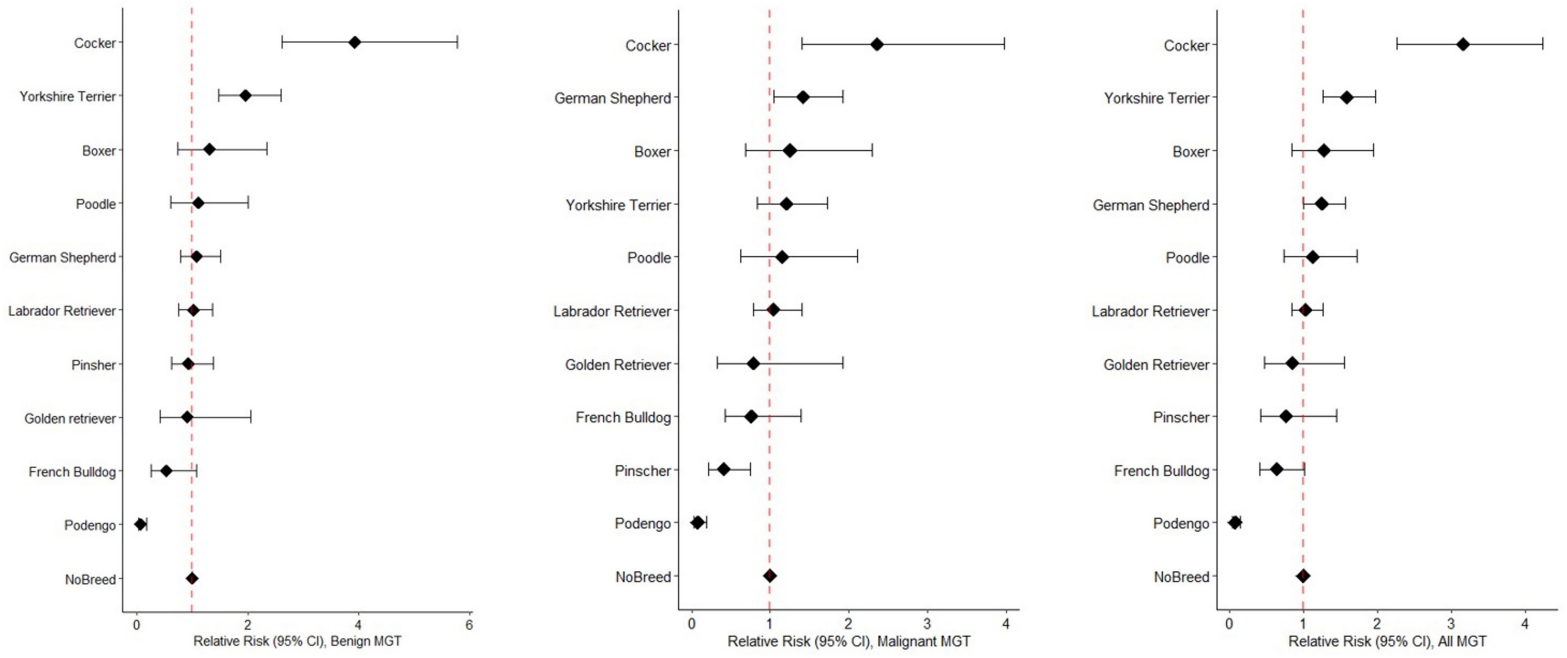
Forest plots illustrating Relative Risks benign MGT **(left)**, malignant MGT **(middle)**, and all MGT **(right)**, sorted by RR values, across different breeds.

Figure 3 displays the distinct risk profiles of benign and malignant tumors across breeds. Cockers have a three-fold higher risk of developing MGT compared to non-breed dogs (RR = 3.2, 95%CI 2.27–4.24). In terms of benign MGT, Cockers (RR = 3.9, 95%CI 2.61–5.78, *p*-value < 0.001) and Yorkshire Terriers (RR = 2.0, 95%CI 1.47–2.60, *p*-value < 0.001) exhibit the highest RRs (Figure 3). Regarding malignant MGT, Cockers and German Shepherds have the highest RRs (RR = 2.4, 95%CI 1.40–3.98, *p*-value = 0.001; RR = 1.4, 95%CI 1.05–1.92, *p*-value < 0.05, respectively). Interestingly, Pinschers demonstrate a protective effect against the development of malignant MGT (RR = 0.4, 95%CI 0.22–0.75, *p*-value = 0.002). It is worth noting that Podengo dogs (a Portuguese breed) have a protective factor against the development of both malignant and benign MGT.

### 3.3 Comparative analysis

In terms of sex, the findings indicate a striking similarity in the distribution between humans and dogs. In the case of canine mMGT, 98.5% (*n* = 528) are found in female dogs, with only a mere 1.5% in males (*n* = 8). Similarly, the analysis of human subjects reveals a higher incidence of breast cancer in women (*n* = 7,584, 99%) compared to men (*n* = 90, 1%).

Regarding the age distribution, Supplementary Figure S1 displays histograms and corresponding Z-scores, revealing two prominent age peaks—one before and one after the mean age. However, when examining the histogram and Z-score of female dogs with mMGT, only a single frequency peak emerges at the mean age.

The Z-score analysis demonstrates that the distributions in both species are strikingly similar, with no significant differences found in the median of Z-scores (Wilcoxon rank sum test, *p*-value = 0.667). However, there are statistically significant differences in variances (F test = 0.895, *p*-value = 0.011) (Supplementary Table S2).

**Figure 4:**
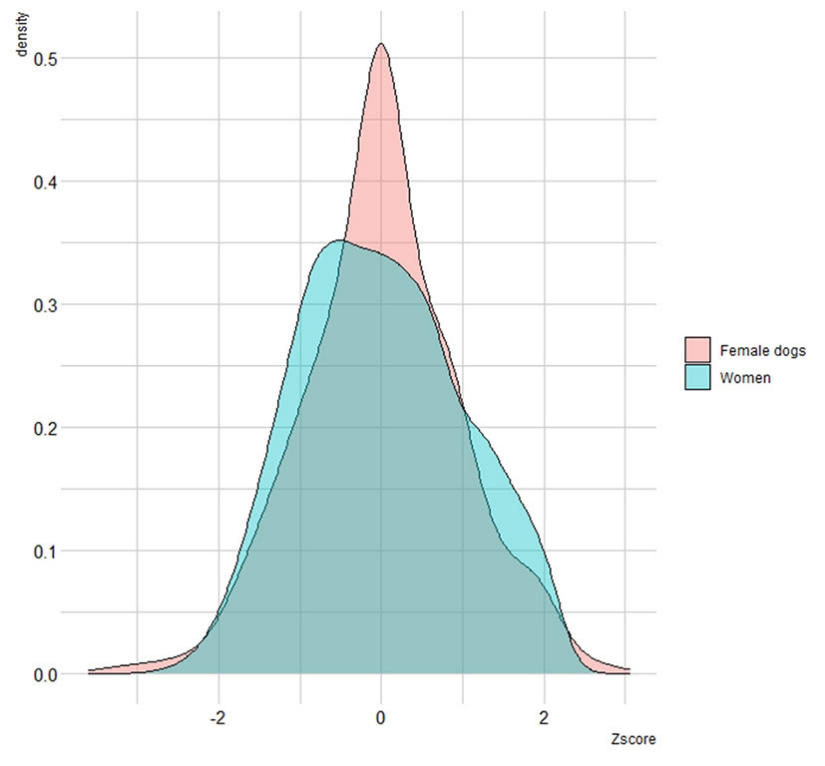
Density plot comparing Z-Scores of ages at diagnosis of women and female dogs.

Figure 4 illustrates a combined density plot of the Z-scores, showcasing a considerable overlap in age distributions between the two species. However, the results of the two-sample K-S test indicate that the distributions of Z-scores for women and female dogs are statistically distinct (K-S = 4.3, *p*-value < 0.001).

### 3.4 Morphology

Among the most frequent morphologies observed in women (Supplementary Table S3), Invasive breast carcinoma (former infiltrating duct carcinoma, NOS (8500/3) accounts for 74.06% (*n* = 5,617). Interestingly, this particular morphology is present in only 0.19% (*n* = 1) of female dogs (Supplementary Table S3). It is worth noting that lobular carcinoma, NOS (8520/3), infiltrating duct mixed with other types of carcinomas (8523/3), and infiltrating duct and lobular carcinoma (8522/3) are not documented in Vet-ICD-O-canine-1, making them incomparable (Supplementary Table S3).

When comparing specific morphologies (Supplementary Table S3), invasive micropapillary carcinoma of the breast (8507/3) accounts for 0.55% (*n* = 42) of cases in women, whereas it represents 1.90% of mMGT cases in female dogs (*n* = 10). In women, intraductal papillary adenocarcinoma with invasion (8503/3) corresponds to 0.46% (*n* = 35) of cases, whereas in female dogs, it accounts for 0.95% (*n* = 5) (Supplementary Table S3).

Shifting the focus to the most frequent morphologies observed in female dogs (Supplementary Table S3), complex carcinoma (8983.1, *n* = 202, 38.33%) lacks a corresponding morphology in women. Tubular carcinoma (8211/3) represents 21.82% (*n* = 115) of mMGT cases, whereas in women, it constitutes only 0.37% (*n* = 28). It is noteworthy that Solid carcinoma (8230/3), observed in 14.61% (*n* = 77) of female dogs, is not reported in women. Additionally, both species exhibit low occurrences of inflammatory carcinoma (8530/3) (0.57% in female dogs and 0.08% in women).

### 3.5 Geographic distribution

The distribution of WBC cases by municipalities displayed at Supplementary Table S4 shows that Porto accounted with 18.5% (*n* = 1,403), Vila Nova de Gaia for 17.1% (*n* = 1,297) of cases, followed by and Matosinhos with 11.7% (*n* = 886). Regarding the distribution of female dogs with mMGT, the municipality of Porto had 24.3% (*n* = 128) of cases, followed by Vila Nova de Gaia with 16.1% (*n* = 85), and Matosinhos with 14.4% (*n* = 76) (Supplementary Table S5).

**Figure 5:**
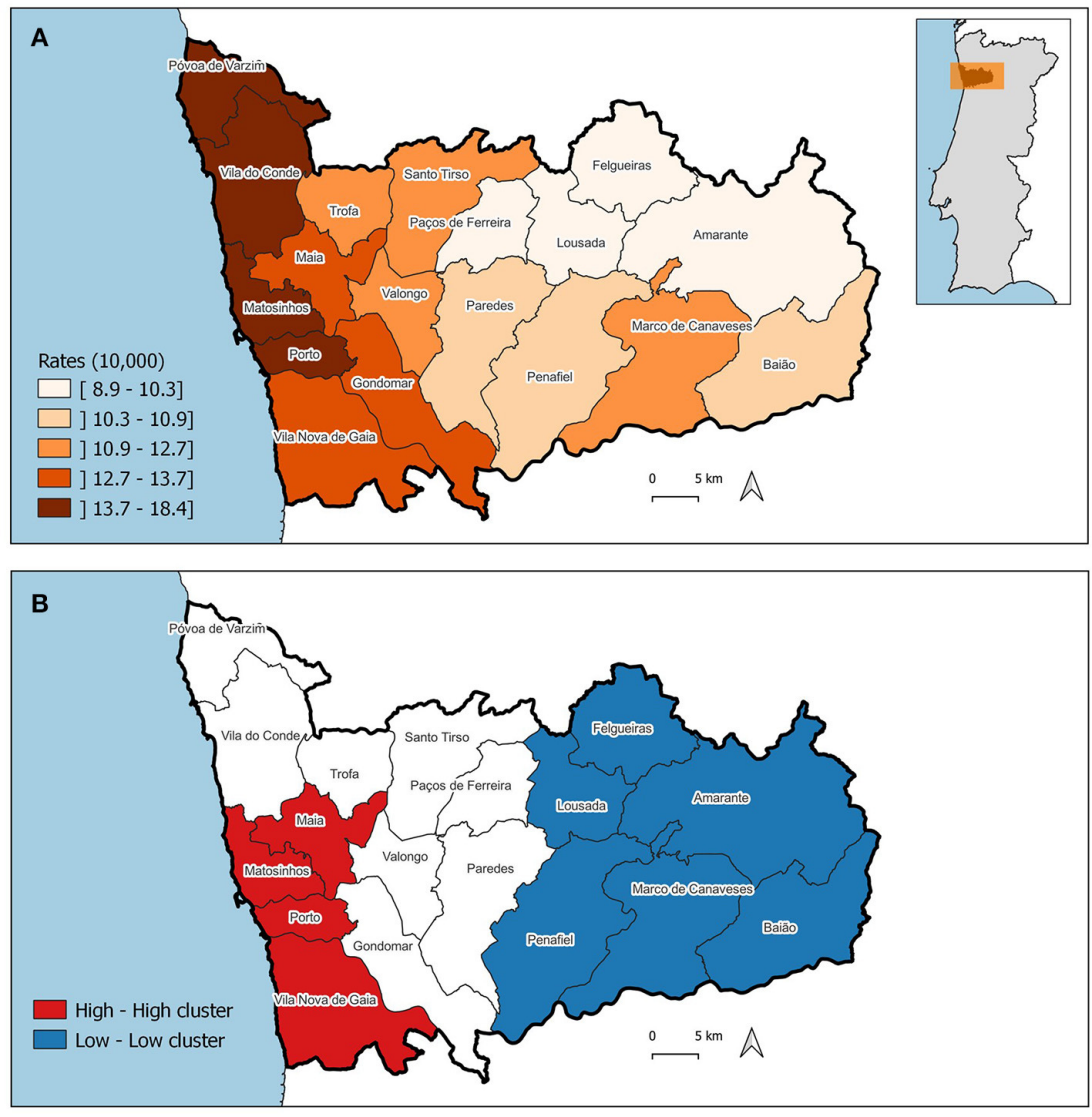
Geographical distribution of the age-standardized incidence risk (ASIR) of women breast cancer per 10,000 women by municipality in the district of Porto. **(A)** ASIR per municipalities. **(B)** LISA cluster map highlighting geographical clusters.

**Figure 6:**
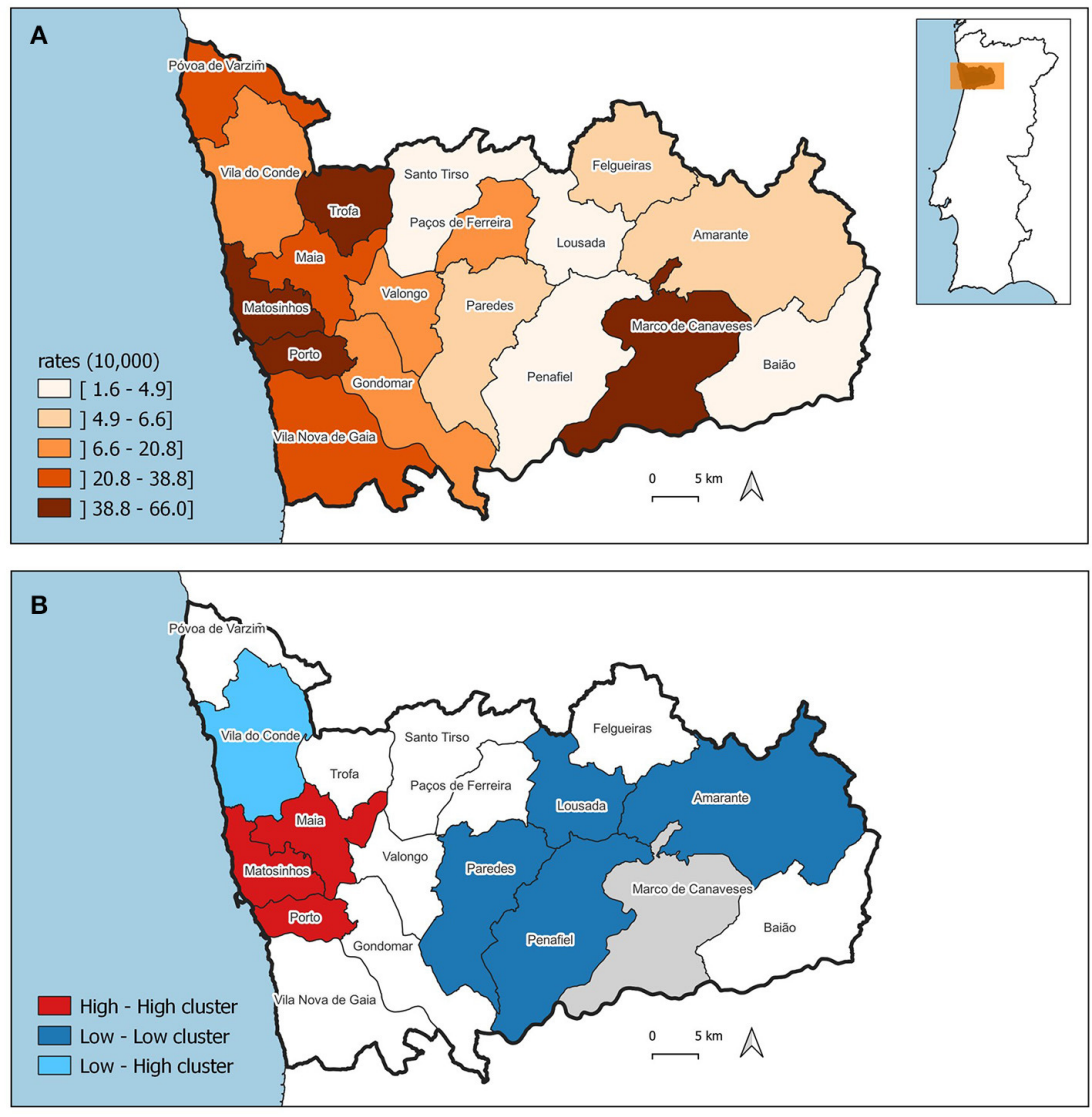
Geographical distribution of age-standardized incidence risk (ASIR) of malignant mammary gland tumors per 10,000 female dogs by municipality in the district of Porto. **(A)** ASIR per municipalities. **(B)** LISA cluster map highlighting geographical clusters.

The pattern of the ASIR of WBC and mMGT is significantly clustered, being the Moran's I 0.656 (*p* = 0.001) and 0.443 (*p* = 0.001), respectively. The ASIR of WBC and mMGT across municipalities are depicted on the maps from Figures 5A, 6A. These maps show that ASIR are higher in coastal and highly urbanized municipalities and lower in municipalities located in the inner land. Several clusters of high rates were identified, and we found that many LISA spatial clusters of WBC and mMGT overlap (Figures 5B, 6B). In WBC, clusters of high rates were found in Maia, Matosinhos, Porto, and Vila Nova de Gaia (Figure 5B), and, in mMGT, they were found in Maia, Matosinhos and Porto (Figure 6B).

Moreover, we found that municipalities with high ASIR of cancer in women tend to be surrounded by municipalities with high ASIR among female dogs. The result of the bivariate Moran's I was moderate and statistically significant (0.434, *p* = 0.001).

The correlation analysis show, at first, a coefficient (R) of 0.69 and a coefficient of determination (R^2^) of 0.47 (*p*-value < 0.01). Based on the results displayed at Supplementary Table S6 and observations from Figure 6A, it is evident that the data from the municipality of Marco de Canaveses deviates from the surrounding ASIRs. To quantify this deviation, a Cook's distance was calculated (c = 1.29), indicating that Marco de Canaveses' ASIR stands out as an outlier. Subsequently, a second correlation analysis was conducted, which revealed an improved correlation coefficient of 0.85 and an R^2^ value of 0.73 (*p*-value < 0.01).

**Figure 7:**
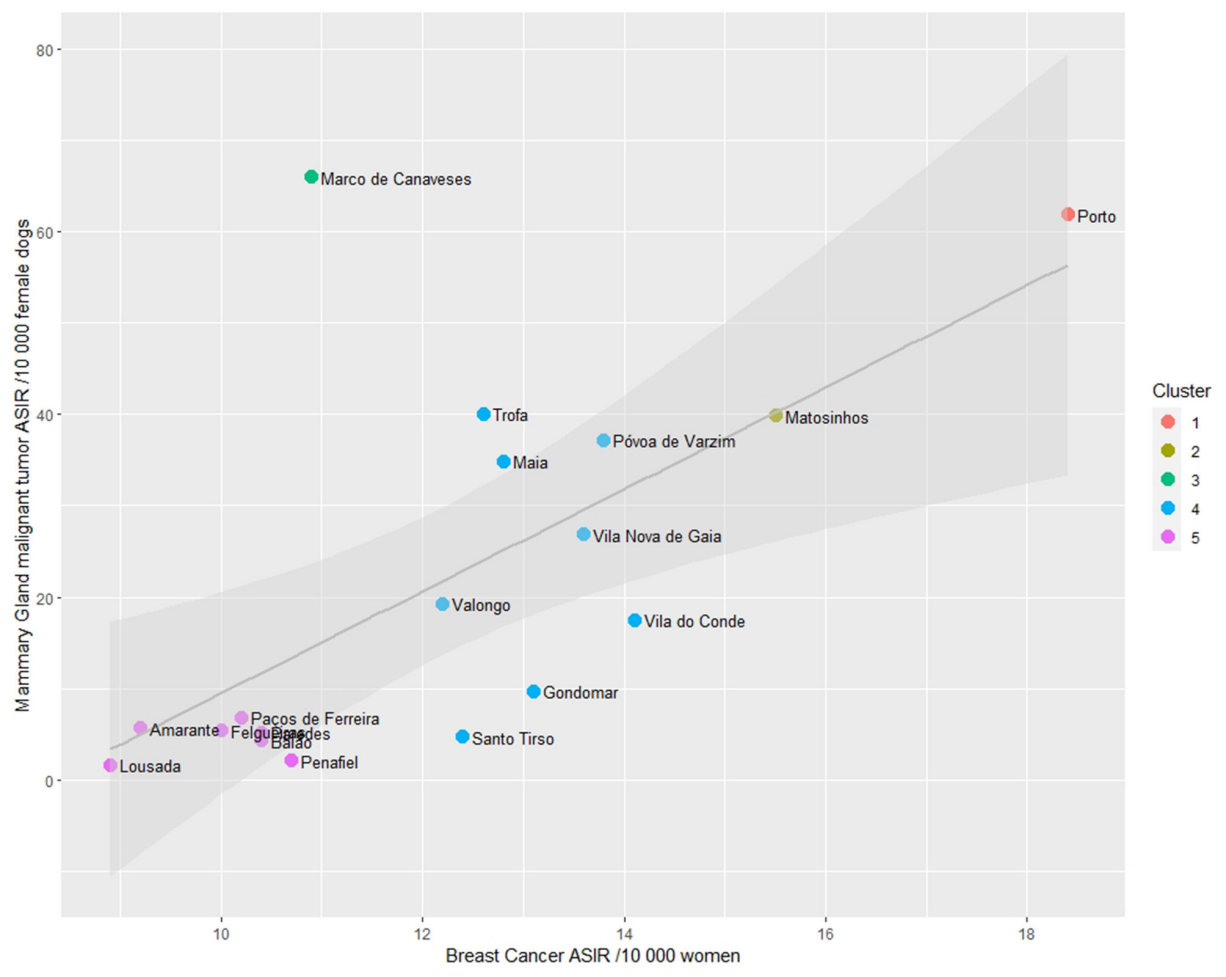
Scatterplot of Age-standardized Incidence Risk (ASIR) of women breast cancer and malignant mammary gland malignant tumors (per 10,000) by municipality in the district of Porto and cluster analysis.

Finally, the ASIRs' cluster analysis revealed the presence of five distinct clusters, as depicted in Figure 7. When observing the clusters, it is noticeable that the municipality of Porto, which is the main city of the Porto district and Porto Metropolitan Area, exhibits high ASIRs both in women and female dogs (cluster 1), followed by Matosinhos (cluster 2). The urban municipalities that surround Porto are grouped in cluster 4, while the more rural peripheral municipalities are in cluster 5 (showing low ASIR values in both women and female dogs).

## 4 Discussion

This work represents a pioneering effort in approaching Human Breast Cancer (HBC) and Canine Mammary Gland Tumors (MGT) from a One Health perspective and investigating their geographical distribution within Porto District. By studying the epidemiological characteristics of both human and canine mammary cancers in the same geographic area, where analogous environmental factors are at play, it becomes possible to describe and compare remarkably similar features in both species.

In this analysis, notable similarities were observed in the distribution by sex, with a predominance of cases in women and female dogs. Additionally, a similar age pattern was identified. However, when examining tumor morphology using the Vet-ICD-O-canine-1 classification system, it was found that the most frequent morphologies differ between the two species.

In the case of canine MGT, certain breeds exhibited a higher relative risk (such as Cocker and Yorkshire Terriers), while others showed a protective effect (like Pinschers and Podengos) against neoplasia development. Furthermore, the study emphasized the similar geographical hotspots and a strong correlation between the ASIR in the municipalities of Porto District. This finding supports the notion of dogs serving as sentinels for human oncological epidemiology.

Overall, this research sheds light on the shared aspects and differences between HBC and canine MGT, highlighting the potential of cross-species studies to enhance our understanding of cancer in both humans and dogs.

### 4.1 Age

Age is the most prominent known risk factor for breast cancer (BC), and it has been observed that the incidence of BC peaks around the age of menopause in women before gradually declining or remaining steady (1, 2). These findings align with the results obtained in this analysis. Similarly, in dogs, the risk of mammary gland tumors (mMGT) increases significantly with age, particularly in geriatric female dogs, reaching a peak diagnosis between 11 to 13 years of age (3, 4). The mean age at diagnosis was found to be 9.9 years, consistent with recent studies (5).

To compare the age distribution between women with breast cancer (WBC) and female dogs with mMGT, a Z-Score calculation was performed in this study. The analysis revealed overlapping age distributions in both species, which supports findings from previous studies (53).

The findings of this study consistently demonstrate a higher prevalence of breast cancer (BC) and mammary gland tumors (MGT) in women/females compared to men/males, which aligns with previous research (39, 40). The influence of the endocrine environment, characterized by exposure to estrogen and progesterone, has been implicated in the development of MGT (36, 37).

Unfortunately, due to the lack of available information in many cases, the reproductive status (spayed/neutered) of female dogs could not be considered in this study. However, it is important to emphasize the significance of including this data in future research. Collaborative efforts involving Veterinary Laboratories and clinicians should be promoted to collect information on the reproductive status of dogs and the age at which they were neutered. Incorporating this aspect will contribute to a more comprehensive understanding of the relationship between reproductive factors and the development of MGT in female dogs.

### 4.2 Topography

Among women, one of the most frequently observed topographies in this analysis was the upper-outer quadrant (UOQ), which is consistent with findings from other studies (54). The high incidence of UOQ involvement can be attributed to the greater proportion of epithelial tissue present in this region, which serves as the primary site for breast cancer development (54).

In the case of dogs, the analysis was hindered by the fact that a majority of the cases were classified as mammary gland, NOS (not otherwise specified). This limitation may be attributed to the absence of site-specific information provided by clinicians. Nonetheless, previous studies have reported that the caudal and inguinal abdominal mammary glands are the most commonly affected sites (55).

### 4.3 Morphology

The distribution of women breast cancer (WBC) by morphology revealed that Invasive breast carcinoma of no special type was the most common type of tumor, followed by lobular carcinoma, NOS, which aligns with previously published literature (56, 57). In female dogs, the most frequent type of mammary gland tumor (mMGT) was complex carcinoma, followed by tubular carcinoma and solid carcinoma, confirming findings reported in the literature (8).

Interestingly, there were specific morphologies where a correspondence was observed between WBC and female mMGT in dogs, particularly in cases of invasive micropapillary carcinoma of the breast (8507/3) and intraductal papillary adenocarcinoma with invasion (8503/3). Invasive micropapillary carcinoma (IMPC) is known to be one of the most aggressive types of breast cancer (58). Previous studies investigating canine IMPC have demonstrated several similarities to the corresponding human disease (14, 59).

Furthermore, both women and female dogs exhibited a low incidence of inflammatory carcinoma, which is consistent with findings from previous studies (60, 61). These observations suggest that canine MGT may serve as a suitable model for comparative pathological studies with human breast cancer (62).

These observations suggest that canine mammary gland tumors (MGT) could serve as a suitable model for comparing pathological aspects with human breast cancer (HBC) (62). The utilization of Vet-ICD-O-canine-1, the veterinary equivalent of ICD-O-3.2, was instrumental in enabling this analysis and serves as an essential tool for comparative oncology studies.

A notable aspect to mention is that more than half of the MGT cases reported in this study among female dogs were benign. Similar to previous publications, complex adenoma and benign mixed tumors were the predominant forms of benign tumors (63). Interestingly, benign mammary tumors are also prevalent throughout a woman's lifetime, ranging from early reproductive years to the postmenopausal phase, highlighting the potential health concern they pose for a significant number of women (64–69).

Despite their prevalence, the etiology and risk factors associated with benign mammary tumors have not been extensively studied, despite an increasing incidence of these tumors detected through population-based mammographic screening (70). The exact incidence rates remain unknown, and previous studies on benign mammary tumors have often been limited in scale and inconsistent in their classification of pathological disease. Consequently, there is a lack of up-to-date studies on incidence rates, and the establishment of risk factors has been limited (64, 65, 71).

Importantly, a history of certain benign tumors is recognized as a risk factor for breast cancer, with benign proliferative disease, with or without atypia, increasing the risk approximately 4-fold and 2-fold, respectively (70, 71). Given that benign breast diseases in women, similar to benign MGT in female dogs, are significant risk factors for breast cancer, future investigations exploring possible associations could enhance the role of the canine model in understanding breast cancer development and progression.

We must emphasize that the critical analysis conducted here was based on ICD-O-3.2, so it may appear somewhat out of context with respect to the updates included in the WHO Classification−5th edition (2019), which are still in need of coding. As an example, we can mention the case of complex carcinoma, which is not included in the ICD-O-3.2.

Another aspect that needs to be noteworthy is that the morphological types of infiltrating duct and lobular carcinoma are not documented in VET-ICD-O-canine-1, preventing their comparison with human counterparts. Although pleomorphic lobular carcinomas were previously described in canine mammary tumors (72–74), they were not included in the first edition of VET-ICD-O-canine-1, highlighting the need for their inclusion in a future update of this classification system.

### 4.4 Breed

Breed has been identified as a risk factor for mammary gland tumors (MGT) in dogs (32, 33). However, the specific breeds reported to be at risk can vary across different studies and geographic locations (32). Breeds such as Poodles (toy and miniature), Spaniels, Pointers, German Shepherds, Maltese Terriers, Yorkshire Terriers, and Dachshunds have all been associated with a predisposition to MGT (33). This variation in breed susceptibility could be influenced by the overpopulation of certain breeds, such as Labrador Retrievers and German Shepherds.

In the present analysis, non-breed dogs were the most affected, followed by Labrador Retrievers, Yorkshire Terriers, and German Shepherds. However, one notable achievement of this study was the calculation of the incidence risk (IR) per breed, which helped mitigate bias stemming from uncertainties in breed population. Consequently, in Porto, Cocker and Yorkshire Terriers were found to be the most affected breeds by MGT, while Pinschers and Podengos demonstrated a protective effect against the development of malignant MGT.

A study conducted in Japan reported a lower incidence of malignancy in mammary tumors among small breed dogs (75), which aligns with our findings showing that purebred dogs like Pinschers had a protective effect against the development of malignant MGT.

These breed-specific age differences in cancer onset may inform future screening programs targeting specific breeds for early cancer detection. The variation in the incidence of mammary tumor risk between breeds suggests a significant heritable genetic component to the disease in dogs (76).

### 4.5 Geographical distribution

The results of this study reveal a strong correlation between the ASIRs of WBC and female dogs mMGT in the Porto district. This finding further emphasizes the potential value of dogs as sentinels for studying human oncological epidemiology. This observation aligns with a recent study that demonstrated a close geographical association, at the municipality level, between human and canine non-Hodgkin's lymphoma (NHL) cases in the Porto region (77).

The similarities between the geographies of WBC and female dogs mMGT in the Porto district suggest that there may exist common environmental determinants, related with the physical environment [e.g., air pollution (78), green space (79)], the social environment [e.g., deprivation (80)], and/or access to healthcare [e.g., geographical accessibility to health services (81)]. These factors should be further investigated in future studies.

It is important to note the elevated incidence risk of female dogs mMGT in the municipality of Marco de Canaveses. One hypothesis suggests that the limited availability of veterinary centers in neighboring regions may funnel cancer cases from surrounding cities into this municipality.

### 4.6 Limitation and bias

One of the limitations of this study is the unavailability of owner postal codes, instead relying on the postal code of the veterinary practice owners' attend to. However, according to a recent study (82), the distance to the veterinary hospital was found to be the main contributing factor for estimating the catchment area (CA), which would reduce the geographical bias. Additionally, to mitigate this bias, the analysis was performed at the municipality level. It is worth noting that dogs have more restricted daily mobility and migration compared to humans, resulting in higher levels of exposure to potentially hazardous environmental risk factors associated with particular locations. Dogs may therefore offer a less biased means of identifying risk environments which would probably strengthen their role as sentinels in investigating human health challenges (83, 84).

Another potential source of bias is related to the data recorded in Vet-OncoNet and SIAC. Vet-OncoNet receives data from confirmed diagnoses by pathology laboratories. Since cancer registration in animals is not mandatory, there is an under-notification of cancer cases in dogs, due to cases that go undiagnosed or that are not sent to the laboratories, and also cases that never reach veterinarians. Despite this data gap, the information presented by Vet-OncoNet can be seen as robust because it represents the activities of nearly all laboratories on a national scale. Additionally, there is no evidence that the under-notification bias varies according to a geographic basis, so is reasonable to assume that the bias in the incidence rate is equally distributed among the municipalities.

There could also exist some under the registry of animals in SIAC, however since this is mandatory since 2018 it is assumed a homogeneous coverage over the territory. Mandatory registration of animals on the SIAC platform makes it an excellent and reliable source for the canine population in Portugal.

Regarding the data collection periods, these were selected based on records' availability. Vet-OncoNet began data collection in 2019, with data available up to the preparation of this study in 2022. RORENO data was only available up to the year 2015, and it was decided to work with data from 2010 onwards. The authors are aware that for better comparison among both population exposure, it would be ideal to have the canine data at least 10 years prior to the period in women. Unfortunately, this is not currently possible. The evidence obtained in this study further emphasizes the importance of maintaining active animal cancer registries, which will enable future studies with greater temporal and geographical accuracy.

Further epidemiological studies should be conducted to investigate carcinogenic factors and explore the genetic and epigenetic factors influencing the study area.

## 5 Conclusions

Dogs spontaneously develop MGT and exhibit striking similarities in epidemiological and clinical characteristics to HBC. Leveraging tools such as human and companion animal oncology registries in Portugal and neoplasm classification systems (ICD-O-3.2 and Vet-ICD-O-canine-1), we conducted a comparative analysis. This study revealed similarities in the distribution by sex and a similar age pattern. However, there are some differences in tumor morphology between the two species. Notably, certain breeds of dogs show a higher relative risk while others exhibit a protective effect.

Significantly, this study underscored a strong correlation and geographical similar patterns between the incidence risks of breast cancer and female dogs' malignant mammary gland tumors in the Porto district.

As both species increasingly share common environments and similar exposomes, these findings support the hypothesis that dogs could serve as valuable sentinels for human oncological epidemiology.

## Data availability statement

The original contributions presented in the study are included in the article/Supplementary material, further inquiries can be directed to the corresponding author.

## Ethics statement

The studies involving humans were approved by Ethics Committee of the Portuguese Institute of Oncology (IPO) in Porto with reference 160/2021. The studies were conducted in accordance with the local legislation and institutional requirements. Written informed consent for participation was not required from the participants or the participants' legal guardians/next of kin in accordance with the national legislation and institutional requirements.

## Author contributions

PC: Conceptualization, Data curation, Formal analysis, Investigation, Methodology, Writing—original draft, Writing—review & editing. JN-R: Conceptualization, Funding acquisition, Methodology, Project administration, Resources, Supervision, Writing—review & editing. IA: Investigation, Writing—review & editing. FQ: Investigation, Validation, Writing—review & editing. MS: Data curation, Formal analysis, Methodology, Visualization, Writing—review & editing. AR: Data curation, Validation, Visualization, Writing—review & editing. KP: Conceptualization, Data curation, Formal analysis, Methodology, Supervision, Validation, Visualization, Writing—original draft, Writing—review & editing.

## Footnotes

^1^ www.siac.vet
