# Supplementary material for: Comparative epidemiological study of breast cancer in humans and canine mammary tumors: insights from Portugal
Source: Front Vet Sci. 2023 Nov 30;10:1271097. doi: 10.3389/fvets.2023.1271097 (PMC10720630; doi:10.3389/fvets.2023.1271097)
Supplement: Supplementary file 1 [file Data_Sheet_1.docx]

Supplementary Material

Comparative Epidemiological Study of Breast Cancer in Humans and Canine Mammary Tumors: Insights from Portugal

Paulo Tiago Carvalho ^1^, João Niza-Ribeiro ^1, 2, 3^, Irina Amorim^4^, Felisbina Queiroga ^5, 6, 7^, Milton Severo^2, 3^, Ana Isabel Ribeiro ^2, 3, 8^ and Katia Pinello^1, 2, 3*^

*** Correspondence:** Corresponding Author: kcpinello@icbas.up.pt

# Supplementary tables

**Table S1:** Age distribution of canine MGT by main breeds (n>10)

| **Breed** | **n (%)** | **Mean (SD)** | **Median (IQR)** |
| --- | --- | --- | --- |
| No-breed  Labrador retriever  Yorkshire terrier  German shepherd  Cocker  Pinscher  Boxer  Poodle  French bulldog  Golden retriever  Podengo | 572 (50.2)  103 (9.0)  88 (7.7)  86 (7.5)  41 (3.6)  37 (3.2)  23 (2.0)  22 (1.9)  19 (1.7)  11 (1.0)  10 (0.9) | 9.9 (2.9) ^b^  10.4 (2.4) ^b^  9.3 (2.0) ^ab^  9.1 (2.3) ^ab^  9.6 (2.4) ^b^  9.4 (2.5) ^ab^  8.5 (2.3) ^ab^  10.0 (2.4) ^b^  7.2 (2.3) ^a^  9.9 (2.3) ^ab^  8.1 (3.1) ^ab^ | 10.0 (4.0)  10.0 (3.0)  9.0 (2.7)  9.0 (4.0)  10.0 (3.0)  10.0 (3.0)  8.0 (2.7)  9.0 (2.0)  7.0 (2.7)  9.5 (3.7)  8.0 (2.5) |

Letters indicates the results of ANOVA followed by Tukey test, p<0.05.

**Table S2**: Descriptive analysis of women and female dogs Z-Scores

| **Z-Score** | **Mean (SD)** | | **IQR** | **0%** | **25%** | **50%** | **75%** | **100%** | **N** | **Variance** |
| --- | --- | --- | --- | --- | --- | --- | --- | --- | --- | --- |
| **Women** | 0.00030 | (1.001) | 1.43 | -2.64 | -0.85 | -0.13 | 0.58 | 2.01 | 7584 | 1.001** |
| **Female Dogs** | 0.00022 | (0.947) | 1.11 | -3.60 | -0.64 | 0.00 | 0.47 | 3.06 | 1115 | 0.897 |

**Table S3:** Distribution of WBC and female dogs mMGT morphologies, according to ICD-O-3.2 and Vet-ICD-O (ordered by ICD-O-3.2 reference code).

| **Women Breast Cancer morphology** | **ICDO-3.2** | **n** | **%** | **Female dog mMGT morphology** | **Vet-ICDO** | **n** | **%** |
| --- | --- | --- | --- | --- | --- | --- | --- |
| Neoplasm, malignant | 8000/3 | 97 | 1.28 | Neoplasm, malignant | 8000/3 | 2 | 0.38 |
|  | 8010/2 |  |  | Carcinoma in situ, NOS | 8010/2 | 3 | 0.57 |
| Carcinoma, NOS | 8010/3 | 153 | 2.02 |  |  |  |  |
|  | 8021/3 |  |  | Carcinoma, anaplastic, NOS | 8021/3 | 5 | 0.95 |
| Pleomorphic carcinoma | 8022/3 | 1 | 0.01 |  |  |  |  |
| Papillary carcinoma, NOS | 8050/3 | 9 | 0.12 |  |  |  |  |
| Squamous cell carcinoma, NOS | 8070/3 | 1 | 0.01 | Squamous cell carcinoma, NOS | 8070/3 | 7 | 1.33 |
| Adenocarcinoma, NOS | 8140/3 | 5 | 0.07 |  |  |  |  |
| Adenoid cystic carcinoma | 8200/3 | 4 | 0.05 |  |  |  |  |
| Cribriform carcinoma, NOS | 8201/3 | 6 | 0.08 |  |  |  |  |
| Tubular adenocarcinoma | 8211/3 | 28 | 0.37 | Tubular carcinoma | 8211/3 | 116 | 22.01 |
|  |  |  |  | Solid carcinoma | 8230/3 | 76 | 14.42 |
| Neuroendocrine carcinoma, NOS | 8246/3 | 6 | 0.08 |  |  |  |  |
| Papillary adenocarcinoma, NOS | 8260/3 | 4 | 0.05 |  |  |  |  |
|  | 8263/3 |  |  | Tubulopapillary carcinoma | 8263/3 | 45 | 8.54 |
| Clear cell adenocarcinoma, NOS | 8310/3 | 1 | 0.01 |  |  |  |  |
|  | 8314/3 |  |  | Lipid-rich carcinoma | 8314/3 | 1 | 0.19 |
| Apocrine adenocarcinoma | 8401/3 | 2 | 0.03 |  |  |  |  |
| Mucinous adenocarcinoma | 8480/3 | 104 | 1.37 | Mucinous adenocarcinoma | 8480/3 | 1 | 0.19 |
| Signet ring cell carcinoma | 8490/3 | 1 | 0.01 |  |  |  |  |
| Infiltrating duct carcinoma, NOS | 8500/3 | 5617 | 74.06 | Ductal carcinoma | 8500/3 | 1 | 0.19 |
| Comedocarcinoma, NOS | 8501/3 | 3 | 0.04 | Comedocarcinoma | 8501/3 | 5 | 0.95 |
| Glycogen-rich carcinoma | 8502/3 | 3 | 0.04 |  |  |  |  |
| Secretory carcinoma | 8502/3 | 3 | 0.04 |  |  |  |  |
| Intraductal papillary adenocarcinoma with invasion | 8503/3 | 35 | 0.46 | Intraductal papillary carcinoma | 8503/3 | 5 | 0.95 |
| Encapsulated papillary carcinoma with invasion | 8504/3 | 19 | 0.25 |  |  |  |  |
| Invasive micropapillary carcinoma of breast | 8507/3 | 42 | 0.55 | Invasive micropapillary carcinoma | 8507/3 | 10 | 1.90 |
| Medullary carcinoma, NOS | 8510/3 | 17 | 0.22 |  |  |  |  |
| Atypical medullary carcinoma | 8513/3 | 3 | 0.04 |  |  |  |  |
| Duct carcinoma, desmoplastic type | 8514/3 | 1 | 0.01 |  |  |  |  |
| Lobular carcinoma, NOS | 8520/3 | 707 | 9.32 |  |  |  |  |
| Infiltrating ductular carcinoma | 8521/3 | 53 | 0.70 |  |  |  |  |
| Infiltrating duct and lobular carcinoma | 8522/3 | 204 | 2.69 |  |  |  |  |
| Infiltrating duct mixed with other types of carcinoma | 8523/3 | 341 | 4.50 |  |  |  |  |
| Infiltrating lobular mixed with other types of carcinoma | 8524/3 | 18 | 0.24 |  |  |  |  |
| Polymorphous adenocarcinoma | 8525/3 | 1 | 0.01 |  |  |  |  |
| Inflammatory carcinoma | 8530/3 | 6 | 0.08 | Inflammatory carcinoma | 8530/3 | 3 | 0.57 |
| Paget disease, mammary | 8540/3 | 22 | 0.29 |  |  |  |  |
| Paget disease and intraductal carcinoma of breast | 8543/3 | 3 | 0.04 |  |  |  |  |
| Acinar cell carcinoma | 8550/3 | 1 | 0.01 |  |  |  |  |
| Adenosquamous carcinoma | 8560/3 | 2 | 0.03 | Adenosquamous carcinoma | 8560/3 | 8 | 1.52 |
|  | 8562/3 |  |  | Carcinoma and malignant myoepithelioma | 8562/3 | 3 | 0.57 |
| Adenocarcinoma with spindle cell metaplasia | 8572/3 | 1 | 0.01 |  |  |  |  |
| Adenocarcinoma with neuroendocrine differentiation | 8574/3 | 1 | 0.01 |  |  |  |  |
| Metaplastic carcinoma, NOS | 8575/3 | 31 | 0.41 |  |  |  |  |
| Sarcoma, NOS | 8800/3 | 1 | 0.01 | Sarcoma, NOS | 8800/3 | 3 | 0.57 |
| Giant cell sarcoma | 8802/3 | 1 | 0.01 |  |  |  |  |
|  |  |  |  | Undifferentiated sarcoma | 8805/3 | 2 | 0.38 |
|  |  |  |  | Fibrosarcoma, NOS | 8810/3 | 2 | 0.38 |
| Liposarcoma, NOS | 8850/3 | 1 | 0.01 |  |  |  |  |
| Dedifferentiated liposarcoma | 8858/3 | 1 | 0.01 |  |  |  |  |
| Leiomyosarcoma, NOS | 8890/3 | 1 | 0.01 |  |  |  |  |
|  | 8940/3 |  |  | Mixed carcinoma, NOS | 8940/3 | 5 | 0.95 |
| Carcinosarcoma, NOS | 8980/3 | 2 | 0.03 | Carcinosarcoma, NOS | 8980/3 | 11 | 2.09 |
| Myoepithelial carcinoma | 8982/3 | 1 | 0.01 | Malignant myoepithelioma | 8982/3 | 3 | 0.57 |
|  |  |  |  | Complex carcinoma | 8983.1/3 | 202 | 38.33 |
| Phyllodes tumor, malignant | 9020/3 | 4 | 0.05 |  |  |  |  |
| Hemangiosarcoma | 9120/3 | 4 | 0.05 |  |  |  |  |
|  |  |  |  | Osteosarcoma, NOS | 9180/3 | 4 | 0.76 |
| Malignant lymphoma, non-Hodgkin, NOS | 9591/3 | 1 | 0.01 |  |  |  |  |
| Hodgkin lymphoma, NOS | 9650/3 | 1 | 0.01 |  |  |  |  |
| Malignant lymphoma, small B lymphocytic, NOS | 9670/3 | 1 | 0.01 |  |  |  |  |
| Diffuse large B-cell lymphoma, NOS | 9680/3 | 4 | 0.05 |  |  |  |  |
| Burkitt lymphoma, NOS | 9687/3 | 1 | 0.01 |  |  |  |  |
| Marginal zone B-cell lymphoma, NOS | 9699/3 | 3 | 0.04 |  |  |  |  |
| Myeloid sarcoma | 9930/3 | 1 | 0.01 |  |  |  |  |
| **Total** |  | **7584** | **100.00** |  |  | **527** | **100.00** |

Most frequent BHBC Most frequent mMGT Similar frequencies

**Table S4**: List of the distribution of the women population and WBC cases by municipalities in Porto district, between 2010 and 2015 (N and %).

| **Municipality** | **Women Population** | | **WBC Cases** | |
| --- | --- | --- | --- | --- |
|  | **n** | **%** | **n** | **%** |
| Amarante | 27904 | 3.0 | 154 | 2.0 |
| Baião | 9688 | 1.0 | 61 | 0.8 |
| Felgueiras | 29436 | 3.1 | 172 | 2.3 |
| Gondomar | 87484 | 9.3 | 699 | 9.2 |
| Lousada | 24217 | 2.6 | 118 | 1.6 |
| Maia | 73919 | 7.8 | 561 | 7.4 |
| Marco de Canaveses | 26567 | 2.8 | 167 | 2.2 |
| Matosinhos | 93454 | 9.9 | 886 | 11.7 |
| Paços de Ferreira | 28476 | 3.0 | 165 | 2.2 |
| Paredes | 44421 | 4.7 | 264 | 3.5 |
| Penafiel | 36176 | 3.8 | 220 | 2.9 |
| Porto | 119394 | 12.7 | 1403 | 18.5 |
| Póvoa de Varzim | 33313 | 3.5 | 271 | 3.6 |
| Santo Tirso | 35741 | 3.8 | 280 | 3.7 |
| Trofa | 20272 | 2.1 | 151 | 2.0 |
| Valongo | 51881 | 5.5 | 368 | 4.9 |
| Vila do Conde | 793 | 4.4 | 347 | 4.6 |
| Vila Nova de Gaia | 159096 | 16.9 | 1297 | 17.1 |
| **Total** | 943232 | 100.0 | 7584 | 100.0 |

**Table S5**: List of the distribution of the female dog’s population and female malignant Mammary gland tumors (mMGT) cases by municipalities in Porto district (n and %).

| **Municipality** | **Female Dogs Population** | | **Female mMGT** | |
| --- | --- | --- | --- | --- |
|  | **n** | **%** | **n** | **%** |
| Amarante | 5324 | 3.5 | 3 | 0.6 |
| Baião | 1645 | 1.1 | 0 | - |
| Felgueiras | 4618 | 3.0 | 3 | 0.6 |
| Gondomar | 13487 | 8.8 | 22 | 4.2 |
| Lousada | 4248 | 2.8 | 2 | 0.4 |
| Maia | 11318 | 7.4 | 48 | 9.1 |
| Marco de Canaveses | 4557 | 3.0 | 37 | 7.0 |
| Matosinhos | 12948 | 8.5 | 76 | 14.4 |
| Paços de Ferreira | 4581 | 3.0 | 5 | 0.9 |
| Paredes | 8322 | 5.4 | 4 | 0.8 |
| Penafiel | 7452 | 4.9 | 2 | 0.4 |
| Porto | 14891 | 9.7 | 128 | 24.3 |
| Póvoa de Varzim | 5828 | 3.8 | 32 | 6.1 |
| Santo Tirso | 7094 | 4.6 | 7 | 1.3 |
| Trofa | 4547 | 3.0 | 24 | 4.6 |
| Valongo | 7824 | 5.1 | 25 | 4.7 |
| Vila do Conde | 8803 | 5.8 | 24 | 4.5 |
| Vila Nova de Gaia | 25546 | 16.7 | 85 | 16.1 |
| **Total** | 153033 | 100.0 | 527 | 100.0 |

**Table S6:** Women and female dogs’ age standardized incidence risk (ASIR) of breast cancer and mammary malignant tumors by municipality in Porto district.

| **Municipality** | Age standardized incidence risk **per 10 000** | |
| --- | --- | --- |
|  | Women | Female dogs |
| Amarante  Baião  Felgueiras  Gondomar  Lousada  Maia  Marco de Canaveses  Matosinhos  Paços de Ferreira  Paredes  Penafiel  Porto  Póvoa de Varzim  Santo Tirso  Trofa  Valongo  Vila do Conde  Vila Nova de Gaia  Total | 9.2  10.4  10.0  13.1  8.9  12.8  10.9  **15.5**  10.2  10.4  10,7  **18.4**  13.8  12.4  12.6  12.2  14.1  13.6  13.4 | 5.7  4.3  5.4  9.7  1.6  34.9  **66.0**  **39.9**  6.8  5.2  2.1  **61.9**  37.2  4.7  40.1  19.3  17.5  26.9  25.5 |

# Supplementary figure

| **Age** | | **Z-Score** | |
| --- | --- | --- | --- |
| Women Breast Cancer | Malignant Mammary Gland tumors | Women Breast Cancer | Malignant Mammary Gland tumors |
| 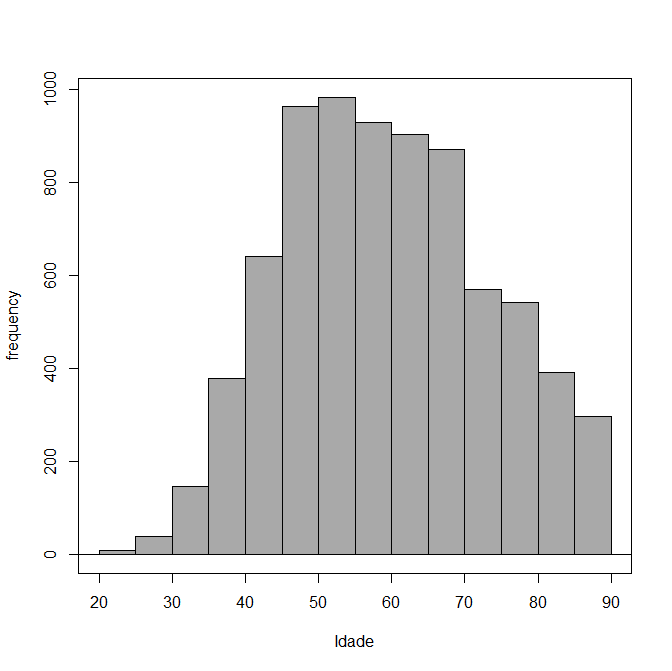 | 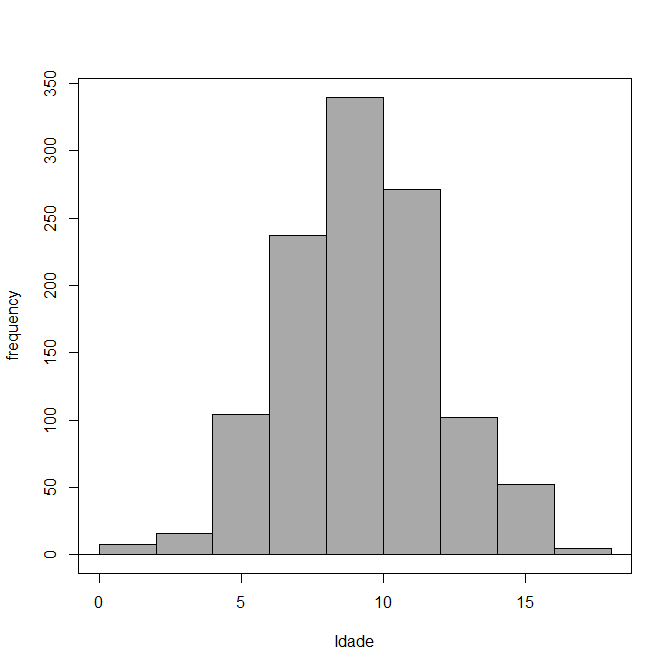 | 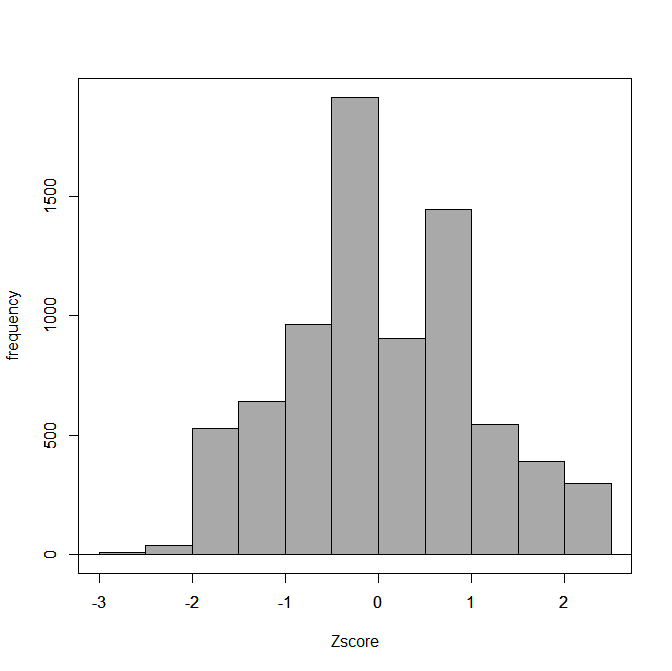 | 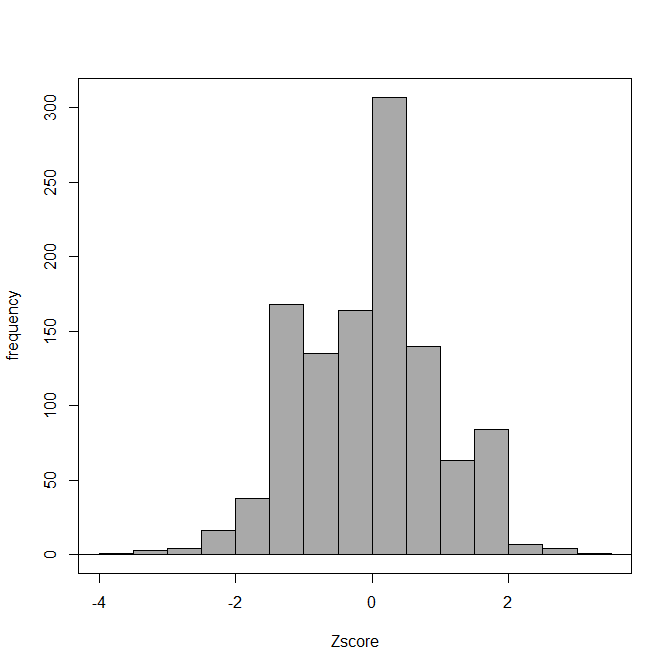 |

**Figure S1:** Histogram of WBC distribution per age (upper-left) and the correspondent Zscore histogram(upper-right), histogram of female dogs’ malignant mammary gland tumores (mMGT) distribution per age (lower-left) and the correspondent Zscore histogram (lower-right).
